# Numerical Study of Complementary Nanostructures for Light Trapping in Colloidal Quantum Dot Solar Cells

**DOI:** 10.3390/nano6040055

**Published:** 2016-03-25

**Authors:** Jue Wei, Qiuyang Xiong, Seyed Milad Mahpeykar, Xihua Wang

**Affiliations:** 1 Department of Electrical and Computer Engineering, University of Alberta, Edmonton, AB T6G 2V4, Canada; weijue@hotmail.com (J.W.); qiuyang@ualberta.ca (Q.X.); mahpeyka@ualberta.ca (S.M.M.); 2 Key Laboratory of Coherent Light and Atomic and Molecular Spectroscopy of Ministry of Education, College of Physics, Jilin University, Changchun 130012, China

**Keywords:** solar cells, nanostructures, light trapping

## Abstract

We have investigated two complementary nanostructures, nanocavity and nanopillar arrays, for light absorption enhancement in depleted heterojunction colloidal quantum dot (CQD) solar cells. A facile complementary fabrication process is demonstrated for patterning these nanostructures over the large area required for light trapping in photovoltaic devices. The simulation results show that both proposed periodic nanostructures can effectively increase the light absorption in CQD layer of the solar cell throughout the near-infrared region where CQD solar cells typically exhibit weak light absorption. The complementary fabrication process for implementation of these nanostructures can pave the way for large-area, inexpensive light trapping implementation in nanostructured solar cells.

## 1. Introduction

In the last few years, colloidal quantum dot (CQD) solar cells have received a great deal of attention due to their potential for large-area, high-throughput, and low-cost manufacturing [1]. Despite all the achievements in CQD synthesis, surface treatment and film deposition technologies [2], the power conversion efficiency of this type of solar cell continues to lag behind traditional silicon solar cells. Because of the lack of long diffusion lengths for photo-generated carriers in CQD films, a CQD film capable of taking advantage of all the incident solar power would be too thick to extract all the generated carriers, leading to an absorption-extraction trade-off [3,4,5]. Light trapping, or effectively increasing optical path lengths in the absorbing material through structuring without any change in light absorbing material’s thickness, is one option to overcome the aforementioned trade-off [6].

Periodic nanostructured gratings have been extensively explored for light trapping in various types of thin-film solar cells and various silicon or metamaterial-based structures have been proposed, such as nanopillar, nanowire, nanohole, and pyramid arrays [7,8,9,10,11,12,13,14]. The downside, however, is that these structures are difficult to fabricate due to their complicated structures or material compositions. Metallic gratings have also been considered for light harvesting enhancement by taking advantage of surface plasmons [15,16,17]. However, parasitic light absorption intrinsic to metallic structures which can compete against useful absorption in light absorbing layer has severely limited the application of metallic nanostructures in photovoltaic devices [18].

Recently, we reported on periodic nano-branch indium-doped tin oxide (ITO) electrodes as diffraction gratings for light absorption enhancement in CQD solar cells [19]. Using numerical simulations, a significant polarization-independent broadband light absorption enhancement was observed for two-dimensional ITO nano-branch gratings and the absorption enhancement was demonstrated to be almost independent of common fabrication flaws in nano-branch structure. On the other hand, current fabrication technologies are unable to implement the fabrication of such nanostructures due to the difficulty of keeping the periodicity of the structure over a large area and also incorporation of CQDs into such porous structure.

In order to be able to apply a periodic nanostructure for practical and tangible light trapping in solar cells, maintaining the periodicity of the structure over a large area is the key requirement. Therefore, the fabrication process used to impose the periodic pattern must be able to produce periodic patterns over large areas and also inexpensive at the same time. Recent advances in large-scale nanofabrication techniques have allowed sophisticated nanostructures to be employed in solar cells and photodetectors with impressive results [20,21]. In this work, in order to fully satisfy the large-area requirement of light trapping structures, we consider two experimentally available nanostructures (nanocavity and nanopillar) for light absorption enhancement in CQD solar cells due to their potential for easy large-area fabrication and CQD incorporation. A facile fabrication process is performed to achieve large-area periodic pattern of the nanocavity and nanopillar structures. The simulation results show that compared to the reference flat structure, our proposed structures can achieve relatively high absorption enhancement in CQD solar cells.

## 2. Results and Discussion

### 2.1. Structure Design

**Figure 1:**
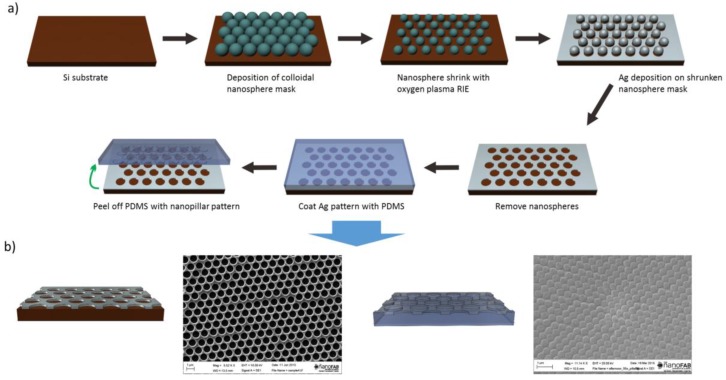
(**a**) The proposed process flow for fabrication of nanocavity and nanopillar arrays. (**b**) Top view scanning electron microscope (SEM) images and cross-sectional schematic of the nanocavity (left) and nanopillar (right) arrays fabricated using the proposed process.

The main reason that nanocavity and nanopillar arrays are proposed as nanostructured electrodes to improve absorption enhancement in PbS CQD solar cells is that such nanostructures can be easily fabricated over large areas by a low-cost pattern transfer method known as nanosphere lithography (NSL). As a proof of concept, PDMS nanopillar and silver nanocavity arrays were fabricated utilizing nanosphere lithography. The proposed process steps for fabrication of nanocavity and nanopillar arrays is presented schematically in Figure 1a. Firstly, colloidal nanosphere mask is deposited on a Si substrate. After nanosphere mask formation, oxygen plasma is employed to shrink the nanospheres to ideal diameter needed for the intended structure through reactive ion etching (RIE). The next step is to deposit the desired material, in this case silver (Ag), on the sample covered by colloidal mask. The final step is to lift off the nanosphere mask, which is usually done in ultrasonic bath with organic solvents such as acetone, after which the nanocavity array is formed on the substrate. In addition to being a standalone light trapping structure, the fabricated nanocavity array can be utilized for fabrication of nanopillar array through PDMS casting and peel off. This is possible because the nanocavity array can act as a mold for formation of nanopillar array. The Scanning Electron Microscope (SEM) images of the cavity and pillar arrays fabricated using the described fabrication process are shown in Figure 1b. We believe a similar procedure with minimal modification can be used to fabricate well-defined ITO nanocavity and nanopillar electrodes. Substituting the silver with ITO in material deposition step will easily lead to ITO nanocavity structure and depositing ITO on top of fabricated PDMS nanopillars can form the desired ITO nanopillars suitable for light absorption enhancement in CQD solar cells.

**Figure 2:**
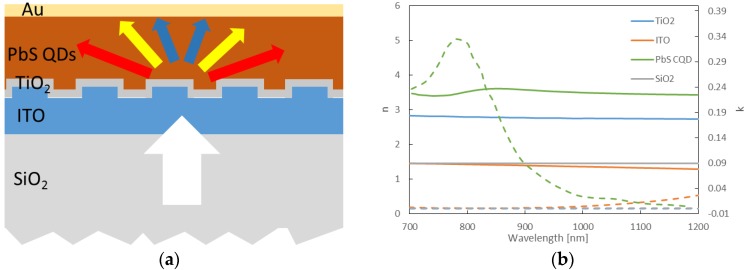
(**a**) Schematic of light diffraction in PbS quantum dot (QD) solar cell with patterned indium-doped tin oxide (ITO) electrode. (**b**) Optical constants of the materials used in the simulation model.

The periodic ITO electrodes proposed in this work are designed for a typical depleted heterojunction CQD solar cell structure. The depleted heterojunction architecture utilizes a TiO_2_ layer as the n-side of the junction and p-type PbS quantum dots as the p-side. The bottom contact to the junction is formed on a glass substrate and consists of a thin transparent conductive ITO layer. The top contact employs a deep work function metal such as gold to collect photo-excited holes and also reflect back any unabsorbed photons into the light absorbing layer. A conformal layer of TiO_2_ with thickness of 50 nm was considered as a layer between ITO electrode and active layer. The designed periodic nanostructures are implemented at the interface between the ITO bottom contact and PbS QDs. Figure 2a shows the schematic of the structure of PbS QD solar cell with patterned ITO electrode used for simulation. As depicted in the figure, when the light is normally incident on ITO diffraction gratings through the transparent substrate, forward diffraction of light can induce light trapping by effectively increasing optical path lengths inside the absorbing material especially for higher diffracted orders supported by the grating structure. Optical constants of the materials used in the simulation model are shown in Figure 2b.

### 2.2. Light Trapping Analysis

**Figure 3:**
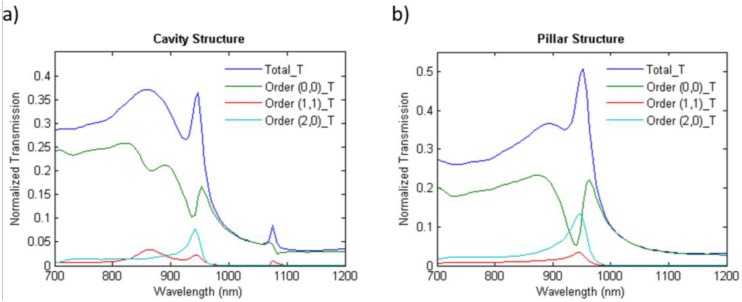
The normalized transmission spectra of simulated patterned ITO structures: (**a**) nanocavity, (**b**) nanopillar. The plot shows the relative power transmitted into different diffracted orders and the net total transmitted power normalized to the simulation source power. Two of the strongest diffracted orders (1,1) and (2,0) are plotted. (0,0) represents the part of incident power not being diffracted by the structures.

Grating far-field projection analysis [22] was firstly used to analyze the diffraction behavior of the proposed periodic grating structures in PbS CQD solar cells. The resulting transmission efficiencies of the simulated patterned structures are illustrated in Figure 3. As is clear from the figure, both cavity and pillar structures demonstrate high transmission efficiency in wavelength range of 700 nm to 900 nm. On the other hand, the amount of transmitted power is not significant beyond 900 nm. This trend, however, is broken for both nanocavity and nanopillar arrays at around the wavelength of 950 nm with a strong increase in the amount of transmitted power. The same behavior is also observed in the case of the cavity array at wavelength of 1080 nm, the intensity of which, however, is not as strong as the peak at 950 nm. This sudden increase in transmitted power can be attributed to the resonant coupling of the incident light into wave guiding modes supported by the PbS CQD layer located adjacent to the ITO grating structures due to the periodic nature of their structure [19]. Although throughout the spectrum, a portion of incident light is not diffracted (shown as the order (0,0)), by paying close attention to the total transmission values and their difference with order (0,0), it is obvious that a significant amount of energy is diffracted into higher orders, especially at resonance wavelengths. This can greatly contribute to light absorption enhancement in CQD layer by increasing the optical path length of the light inside the layer or light trapping through resonant coupling with the incident light [19]. The transmission efficiencies of two of the strongest diffracted orders (1,1) and (2,0) are plotted in Figure 3.

**Figure 4:**
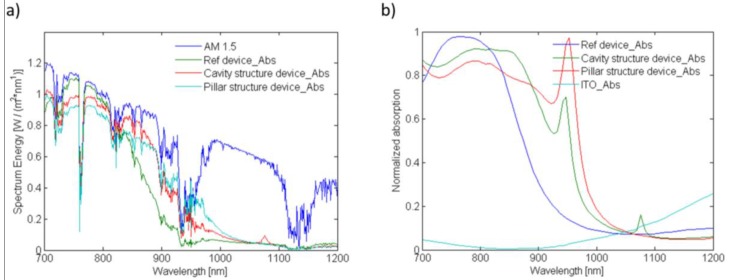
The light absorption spectra for PbS colloidal quantum dot (CQD) layer incorporated into different ITO structures normalized to (**a**) AM1.5G spectra and (**b**) simulation light source. The absorption enhancement for both cavity and pillar structures over the reference flat structure is obvious especially at resonance wavelengths of 950 nm for both structures and 1080 nm for cavity arrays. A slight absorption loss by ITO layer was also observed, as shown in Figure 4b.

Figure 4 depicts the simulated light absorption spectra for PbS CQD layer of the modelled depleted heterojunction solar cell normalized to the AM1.5G solar spectrum (Figure 4a) and simulation light source (Figure 4b). In order to be able to compare the effect of proposed structures on absorption enhancement in PbS CQD layer, a flat ITO layer was considered as the reference. The available power from AM1.5G spectrum is also included in the figure for comparison. As is obvious from the figure, both nanocavity and nanopillar arrays can induce more light absorption in CQD layer than the flat ITO layer within most parts of the near-infrared region. It is also noticeable that both proposed structures have achieved almost perfect absorption in the range of 720 nm to 850 nm by absorbing all the power available from the sun in this range. As for beyond this range, especially in the case of cavity structure, the resonant coupling of the incident light into guided modes supported by CQD layer is the major responsible for strong but narrowband absorption enhancements at resonant peaks, previously predicted by grating projection analysis. This is possible because of the major difference in refractive indices of the CQD layer and the ITO layer which can form an efficient waveguide in the middle of the cell’s structure.

In order to have an overall evaluation for the light absorption enhancement performance of the proposed structures, the average absorption enhancement of the structures over the entire simulated spectrum was measured against the flat reference structure using the following equation:

Absorption Enhancement (%) = (*P_g_* − *P_r_*)/*P_r_* ×100 (1)

where *P_g_* depicts the total power absorbed by the cell with grating structure and *P_r_* denotes the power absorbed by the reference flat structure. According to the equation, the calculated absorption enhancement factors for nanocavity and nanopillar grating structures compared to the flat structure are 15.0% and 13.6%, respectively. This amount of absorption enhancement can significantly boost charge carrier generation and thus short-circuit current density of a CQD solar cell which can ultimately lead to remarkable improvement in power conversion efficiency of the cell.

**Figure 5:**
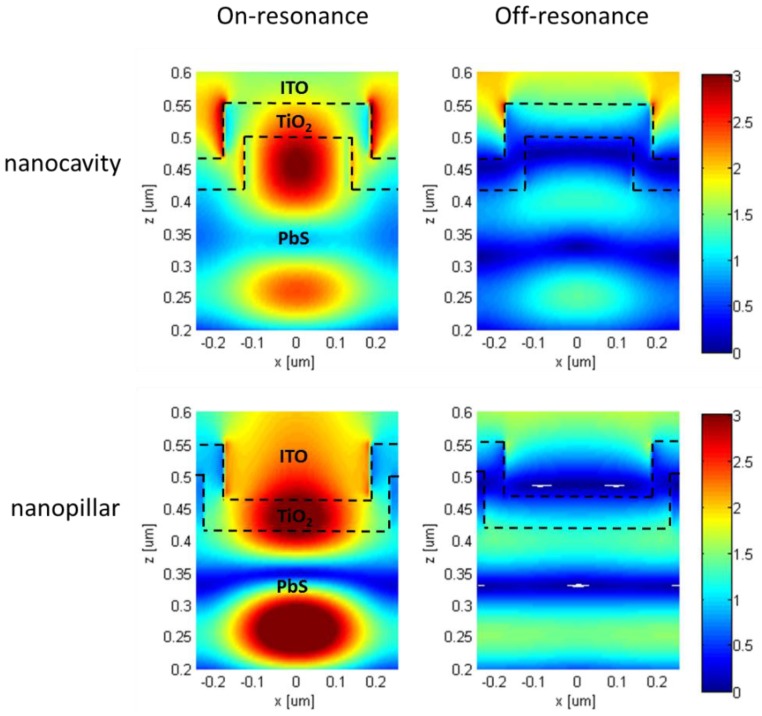
Simulated electric field distributions inside the PbS QDs layer with patterned structures. The hot spots present at resonance wavelengths (950 nm for both structures) with high field intensity indicate strong absorption inside PbS CQD. No hot spots are observed at off resonance wavelengths (1000 nm for both structures) suggesting the importance of resonant coupling of the incident into CQD layer for significant absorption enhancement.

To clearly demonstrate the influence of nanocavity and nanopillar resonance effect on absorption enhancement in CQD layer, the electric field distributions inside the PbS CQD layer with patterned structures were investigated and are shown in Figure 5. The on resonance profiles for nanocavity and nanopillar structures are plotted at wavelength of 950 nm and the off resonant profile wavelength is chosen at 1000 nm for both structures. The on resonance profiles for both structures reveal various absorption hot spots for on-resonance wavelengths where as in the case of off resonance profiles, no hot spot is visible at off-resonance wavelengths. It is obvious that the presence of high intensity E-field spots (hot spots) indicates the occurrence of strong absorption inside PbS CQD layer. In addition, the periodic pattern of the hot spots observed in the obtained profiles discloses the type of resonance to be the guided mode kind usually excited by periodic dielectric nanostructures [6]. The difference in field distribution observed between on and off resonance profiles implies the impressive light trapping performance of the proposed structures at resonance wavelengths. This confirms the superiority of the proposed structures for absorption enhancement in CQD solar cells through resonant coupling of the incident light with supported waveguide modes inside the CQD layer.

## 3. Materials and Methods

### 3.1. Complementary Structure Fabrication

Self-assembly of nanospheres in Si substrate was accomplished by the air/water interface self-assembly process [23]. The etching of deposited nanospheres was done by using a 20 W O_2_ plasma at 5 sccsm oxygen flow for 1 min. E-beam evaporation was used for Silver deposition. The nanosphere mask was removed through sonication in acetone for 10 min. PDMS nanopillars were fabricated by mixing silicone elastomer with curing agent from a Sylgard 184 kit (Dow Corning, Midland, MI, USA) in 10 wt % ratio. The mixture was then degassed in a desiccator for 30 min and was spin-coated on the nanocavity structures at 200 rpm for 30 s. The resulting film was then cured on a hot plate at 80 °C for 2 h after which it was peeled off using a doctor blade. The SEM images were obtained by a Ziess EVO Scanning Electron Microscope (Carl Zeiss, Oberkochen, Germany).

### 3.2. Simulation Methods

The Lumerical FDTD Solutions software (Lumerical Solutions Inc, Vancouver, Canada) was used for simulations in this work. A period of 500 nm was chosen for both cavity and pillar structures which is same as the diameter of nanospheres used for pattern generation. A cavity depth and pillar height of 80 nm and a diameter of 360 nm for both structures was found to be an optimum value. The PbS QD layer was considered to be a quasi-bulk homogeneous film (QDs were not considered as individual particles) without any voids and its thickness (excluding the nanostructure) is chosen to be 300 nm, which is usually considered the maximum thickness for efficient photo-generated carrier collection. The TiO_2_ layer was assumed to be 50 nm thick. Both gold and SiO_2_ glass layers are considered with infinite thickness for ease of modeling. The optimum thickness of ITO layer (excluding the nanostructure) was found to be 500 nm. It should be noted here that the periodic grating structure layer consists of both ITO and PbS materials. The multi-coefficient fitting tool inside the simulation software was utilized to model optical constants of materials from available experimental data [24,25,26,27]. In the case of PbS QDs, the optical constants of commonly used QDs with a bandgap of 1.3 eV were used for simulations.

The light source was considered a planewave source placed inside the substrate (SiO_2_ layer) to simplify the simulations. The wavelength range of 700–1200 nm was chosen as the simulation wavelength span because PbS CQD solar cells are currently in need of absorption enhancement mostly in this region of sunlight [27]. For directions perpendicular to the incident light propagation direction, Bloch boundary conditions and for directions parallel to the light propagation direction, perfectly matched layer (PML) boundary conditions were defined. The amount of absorption inside the CQD layer was measured by placing two power monitors at the either sides of the layer. This configuration can calculate the power flow entering and exiting the layer and thus the power absorbed inside the layer can be obtained by calculating the difference between the measurements from the two monitors. In the case of transmission measurements, all the shown powers are normalized with respect to the power from the light source. For absorption spectra, however, the absorbed powers are normalized to AM1.5G solar spectrum data available from NREL [28].

## 4. Conclusions

In this work, ITO nanocavity and nanopillar diffraction gratings are proposed as light trapping structures in CQD solar cells to realize absorption enhancement and power conversion efficiency improvement. A facile fabrication process is demonstratedfor patterning these periodic nanostructures over a large area which is a critical requirement of practical light trapping structures designed for photovoltaic devices. The simulation results show that both proposed periodic structures can effectively increase the light absorption in CQD layer of the solar cell throughout the near-infrared region where CQD solar cells typically exhibit weak light absorption. The overall absorption enhancement of 15.0% and 13.6% was achieved for nanocavity and nanopillar structures, respectively. It is verified that the two nanostructures are useful for enhancing the efficiency of photovoltaics as large-area, inexpensive light trapping structures.

## Abbreviations

The following abbreviations are used in this manuscript:

CQD: Colloidal Quantum Dot
ITO: Indium-doped Tin Oxide
NSL: Nano Sphere Lithography
RIE: Reactive Ion Etching
SEM: Scanning Electron Microscope
PDMS: Polydimethylsiloxane
FDTD: Finite Difference Time Domain
